# Comparison of survival between unilateral and bilateral breast cancers using propensity score matching: a retrospective single-center analysis

**DOI:** 10.1007/s10549-024-07606-1

**Published:** 2025-01-09

**Authors:** Talar Ozler, Rusen Cosar, Necdet Sut, Dilek Nurlu, Şule Parlar, Sinan Ateş, Mert Hacı Dertli, Yusuf Kavuzlu, Sekip Kavukcu, Mert Chousein, Gokay Yıldız, Nermin Tunçbilek, Muhammet Bekir Hacıoglu, Ebru Tastekin, Sernaz Topaloğlu

**Affiliations:** 1 Department of Radiation Oncology, Faculty of Medicine, Trakya University, Edirne, Turkey; 2 Department of Biostatistics, Faculty of Medicine, Trakya University, Edirne, Turkey; 3 Department of Medical Physics, Faculty of Medicine, Trakya University, Edirne, Turkey; 4 Department of Obstetrics and Gynecology, Faculty of Medicine, Trakya University, Edirne, Turkey; 5 Department of Radiation Oncology, Istanbul Prof. Dr. Cemil Taşçıoğlu City Hospital, Istanbul, Turkey; 6 Department of Radiology, Faculty of Medicine, Trakya University, Edirne, Turkey; 7 Department of Medical Oncology, Faculty of Medicine, Trakya University, Edirne, Turkey; 8 Department of Pathology, Faculty of Medicine, Trakya University, Edirne, Turkey

**Keywords:** Breast Cancer, Synchronous bilateral breast cancer, Metachronous bilateral breast cancer, Bilateral breast cancer, Unilateral breast cancer, Propensity score analysis, Multivariate cox regression analysis with backward stepwise

## Abstract

**Purpose:**

The characteristics of patients with bilateral and unilateral breast cancer at the time of diagnosis or during follow-up have been compared, focusing on the differences in disease-free survival and overall survival between these groups.

**Methods:**

A total of 1,947 patients diagnosed with invasive carcinoma were included in the study. 1876 ​​(96.4%) of our patients had unilateral and 71 (3.6%) had bilateral breast cancer. Among the bilateral breast cancer patients n = 47 were metachronous, while n = 24 were synchronous.

**Results:**

SBBC, which had the lowest OS duration, showed a statistically significant difference compared to MBCC, similar to that observed in unilateral breast cancer (*p* = *0.027*).

**Conclusion:**

The fact that SBBC has the lowest survival rate despite more aggressive treatments should be considered a poor prognostic factor for survival on its own.

## Introduction

Breast cancer screening programs with mammography have led to an increase not only in the incidence of unilateral breast cancer but also in bilateral breast cancer. This is evident from the trend in the literature showing a rise in bilateral breast cancer incidence over the years, ranging from 1.4% to 21% [1–4]. Bilateral breast cancer can present in two ways: as synchronous bilateral breast cancer (SBBC) when diagnosed at the same time as the first tumor or within 3, 6, or 12 months after the initial diagnosis, or as metachronous bilateral breast cancer (MBBC) if diagnosed after a longer period [5–8]. Different time criteria are reported in the literature on this subject. MBBC is more common than SBBC, the incidence of SBBC accounts for 1% to 3% of all breast cancer cases [2, 3].

Although there are studies in the literature investigating the clinicopathological features that predispose to bilateral breast cancer and the effects of these features on disease-free survival and overall survival, their numbers are quite limited [4, 6, 7]. There are also conflicting data regarding whether the development of bilateral breast cancer affects prognostic outcomes [2]. While some series have analyzed that bilaterality negatively impacts overall and disease-free survival [4–8], others have found no significant difference [3, 9, 10]. The effect of bilaterality on survival remains uncertain. Additionally, SBBC (synchronous bilateral breast cancer) is often associated with larger tumor sizes and worse histopathological features. However, some studies in literature report findings that contradict these associations, highlighting ongoing disagreements and inconsistencies in this area of research. However, the increase in the incidence of bilateral breast cancer in recent years will help to conduct more clinical studies on the subject, which will help to clarify its prognostic significance and treatment protocols [2–4, 10–12].

We aim to compare the prognostic factors and survival time of bilateral and unilateral breast cancer patients in our series. To achieve this, we used propensity score analysis to balance the groups in terms of number and prognosis. We also analyzed the prognostic impact of bilaterality and patient or treatment characteristics affecting prognosis.

## Materials and methods

The Human Research Ethics Committee of Trakya University Faculty of Medicine approved the use of patients’ information for the study (TUTF-GOBAEK 2020/334). To use the relevant information, informed consent forms were obtained from patients or the relatives of deceased patients in accordance with the Helsinki Declaration [13]. Following the approval of the institutional review board, the records of 2,086 breast cancer patients who applied to the Departments of Radiation Oncology and Medical Oncology at Trakya University Faculty of Medicine between July 1999 and December 2019 were examined. Data from 127 patients with in situ ductal carcinoma and 12 men among the unilateral breast cancer patients were excluded. A total of 1,947 patients with a diagnosis of invasive carcinoma were included in the study. The following parameters were analyzed for these cases: gender, age, family history, menopausal status, histological types, the location of the tumor at the time of diagnosis, whether bilateral breast cancer was metachronous or synchronous, clinical and pathological TNM stages, grade mentioned in pathology reports, lymphovascular invasion, perineural invasion, skin involvement, surgical margin status, hormone receptors, Ki-67 index, status of extracapsular invasion, type and date of surgery, axillary approach, type of chemotherapy, use of hormone therapy, whether they received radiotherapy, and the type of radiotherapy received. The staging was performed according to AJCC 2017 guidelines [14].

The characteristics of patients with bilateral and unilateral breast cancer at the time of diagnosis or during follow-up have been compared, focusing on the differences in disease-free survival and overall survival between these groups. Prognostic factors contributing to this difference have been investigated.

### Statistical analysis

The statistical analysis of the findings obtained in the study was performed using the SPSS (Statistical Package for Social Sciences) 20.0 program. Numerical results were expressed as mean ± standard deviation, while categorical results were presented as "n". Normality distribution of the numeric variables were tested by the Shapiro-Wilks test. The data were divided into two groups, unilateral and bilateral. Comparisons between the two groups were made using the Student’s T-test for continuous variables and the Pearson Chi-Square test or Fisher’s Exact test for categorical variables. A logistic regression model was used to analyze risk factors associated with bilateral breast cancer. Propensity score matching (PSM) was performed between the unilateral and bilateral breast cancer groups using an optimal matching algorithm (optimum, 1:1) with variables such as axillary nodal status, subgroup, ER status, HER-2 status, and surgical type [15, 16]. Variables that showed significant differences between the two groups or were clinically significant as determined by the Pearson Chi-Square test were used to create propensity scores. After PSM, DFS and OS were calculated using the Kaplan–Meier method. Survival curves were generated using the Kaplan–Meier method, and the significance of survival differences between selected variables was compared using the log-rank test [17]. Univariate Cox regression analysis was used to estimate hazard ratios. Then, multivariate Cox regression analysis with a backward stepwise method was performed to estimate hazard ratios and identify independent prognostic factors [18]. All reported p-values were two-sided, and p-values below 0.05 were considered significant.

## Results

In our study, 1947 patients diagnosed with invasive breast cancer, who received treatment and had follow-up, were included. The median follow-up period was 77.3 months. Of the 1947 patients whose records we reviewed, 1876 (96.4%) had unilateral breast cancer, and 71 (3.6%) had bilateral breast cancer. Among the bilateral breast cancer patients, 66.2% (n = 47) were metachronous, and 33.8% (n = 24) were synchronous. There were no male patients in the bilateral breast cancer group. The average age in the unilateral group was 52 years, while in the bilateral group, it was 52.8 years. A family history of breast cancer was present in 31.5% (n = 595) of the patients in the unilateral group and 32.3% (n = 23) in the bilateral group (Table 1).

**Table 1:** Comparing the clinical, histopathological and treatment features of our patients with unilateral and bilateral breast cancer before and after propensity score analysis

	Pre-propensity score patient characteristics			Post-propensity score patient characteristics		
	Unilateral BC n (%)	Bilateral BC n (%)	p value	Unilateral BC n (%)	Bilateral BC n (%)	p value
Patient Characteristics	1876 (99,40)	71 (0,6)		71	71	
Positive Family History	595 (31,50)	23 (32,40)	0,876	25 (35,20)	23 (32,4)	0,723
Menstrual Status						
Premenopause	746 (39,60)	25 (35,20)	0,537	25 (35,20)	25 (35,20)	1,000
Postmenopause	1130 (60,40)	46 (64,80)		46 (64,80)	46 (64,80)	
Histopathology						
IDC	1548 (82,00)	53 (74,60)	0,119	55 (77,50)	53 (74,60)	0,694
Non-IDC	340 (18,00)	18 (25,40)		16 (22,50)	18 (25,40)	
Quandrant Site						
Medial	383 (20,30)	10 (14,10)		10 (14,10)	10 (14,10)	
Lateral	1136 (60,20)	45 (63,40)	0,507	44 (62,00)	45 (63,40)	0,944
Areola	239 (12,70)	9 (12,70)		9 (12,70)	9 (12,70)	
Multifocal	130 (6,90)	7 (9,90)		8 (11,30)	7 (9,90)	
T Stage						
T1	647 (34,30)	23 (32,40)		5 (7,00)	23 (32,40)	
T2	999 (52,90)	37 (52,10)	0,958	42 (59,20)	37 (52,10)	***<0,001***
T3	145 (7,70)	6 (8,50)		8 (11,30)	6 (8,50)	
T4	97 (5,10)	5 (7,00)		16 (22,50)	5 (7,00)	
N Stage						
N0	832 (44,10)	37 (52,10)		32 (45,10)	37 (52,10)	
N1	521 (27,60)	11 (15,50)	***0,024***	12 (16,90)	11 (15,50)	0,807
N2	361 (19,10)	11 (15,50)		11 (15,50)	11 (15,50)	
N3	174 (9,20)	12 (16,90)		16 (22,50)	12 (16,90)	
Grade						
I	278 (14,70)	7 (9,90)		6 (8,50)	7 (9,90)	
II	915 (48,50)	31 (43,70)	0,200	31 (43,70)	31 (43,70)	0,955
III	692 (36,70)	33 (46,50)		34 (47,90)	33 (46,50)	
LVI Positive	918 (48,60)	36 (50,70)	0,809	32 (45,10)	36 (50,70)	0,502
PNI Positive	362 (19,20)	12 (16,90)	0,759	13 (18,30)	12 (16,90)	0,826
Positive Skin Involvement	117 (6,20)	5 (7,00)	0,800	13 (18,30)	5 (7,00)	***0,044***
Positive Surgical Margin	350 (18,50)	14 (19,70)	0,757	17 (23,90)	14 (19,70)	0,542
ER (+)	1505 (79,70)	48 (67,60)	***0,017***	46 (64,80)	48 (67,60)	0,723
ER (-)	383 (20,30)	23 (32,40)		25 (35,20)	23 (32,40)	
PR (+)	1250 (66,20)	48 (67,60)	0,898	33 (46,50)	48 (67,60)	***0,011***
PR (-)	638 (33,80)	23 (32,40)		38 (53,50)	23 (32,40)	
HER-2 (+)	450 (23,80)	10 (14,10)	***0,063***	10 (14,10)	10 (14,10)	1,000
HER-2(-)	1438 (76,20)	61 (85,90)		61 (85,90)	61 (85,90)	
Kİ67 <%15	474 (25,10)	13 (18,30)	0,211	10 (14,10)	13 (18,30)	
Kİ67 ≥ %15	1414 (74,90)	58 (81,70)		61 (85,90)	58 (81,70)	0,494
Extracapsular Invasion	321 (17,00)	11 (15,50)	0,872	10 (14,10)	11 (15,50)	0,813
Molecular Subtype						
Luminal A	386 (20,40)	8 (11,30)		8 (11,30)	8 (11,30)	
Luminal B	1162 (61,50)	43 (60,60)	***0,004***	39 (54,90)	43 (60,60)	0,893
HER-2 Enriched	130 (6,90)	3 (4,20)		3 (4,20)	3 (4,20)	
Triple Negative	210 (11,10)	17 (23,90)		21 (29,60)	17 (23,90)	
Surgical Type						
BCS	986 (52,20)	29 (40,80)		37 (52,10)	29 (40,80)	
MRM	890 (47,10)	42 (59,20)	***0,088***	34 (47,90)	42 (59,20)	0,213
None Surgical	12 (0,60)	0 (0,00)		0 (0,00)	0 (0,00)	
Axillary Surgery						
SLNB	447 (23,70)	10 (14,10)	**0,067**	39 (54,90)	10 (14,10)	***<0,001***
ALND	1431 (75,80)	61 (85,90)		32 (45,10)	61 (85,90)	
None Surgery	10 (0,50)	0 (0,00)		0 (0,00)	0 (0,00)	
Chemotherapy Time						
Neoadjuvan	217 (11,50)	8 (11,30)	0,903	17 (23,90)	8 (11,30)	***0,005***
Adjuvan	1369 (72,50)	53 (74,60)		53 (74,60)	53 (74,60)	
None	302 (16,00)	10 (14,10)		1 (1,40)	10 (14,1)	
Chemotherapy Type						
AC+Taxanes			***0,068***			***0,018***
FAC/FEC/TAC	836 (4,30)	25 (35,20)		26 (36,60)	25 (35,20)	
CMF	635 (33,60)	26 (36,60)		38 (53,50)	26 (36,60)	
Pertuzumab+ Taxane	35 (1,90)	5 (7,00)		2 (2,80)	5 (7,00)	
None	80 (4,20)	5 (7,00)		4 (5,60)	12 (16,90)	
	302 (16,00)	10 (14,00)		1 (1,40)	0 (0,00)	
Using TAM	737 (39,00)	29 (40,80)		19 (26,70)	29 (40,80)	
Not Using TAM	1151 (61,00)	42 (59,20)	0,432	52 (73,20)	42 (59,20)	***0,039***
Using AI	1154 (61,10)	39 (54,90)		33 (46,50)	39 (54,90)	
Not Using AI	734 (38,90)	32 (45,10)	0,533	38 (53,50)	32 (45,10)	0,595
Receiving Transtuzumab	354 (18,80)	9 (12,70)	0,196	8 (11,30)	9 (12,70)	0,796
Received RT	1694 (89,70)	59 (83,10)	**0,078**	67 (94,40)	59 (83,10)	***0,034***
RT Type						
Only Breast RT	570 (30,20)	20 (28,20)	0,202	24 (33,80)	20 (28,20)	0,102
Locoregional RT	1124 (59,50)	39 (54,90)		43 (60,60)	39 (54,90)	

*BC* Breast cancer, *IDC* Invasive Ductal Carcinoma, *T* Tumour, *N* Nodal, *BCS* Breast Conserving surgery, *MRM* Modified Radical Mastectomy, *SLND* Sentinel Lymph Node Dissection, *ALND* Axillary lymph node dissection, *LVI* Lymphovasculer invasion, *PNI* Perineural invasion, *ER* Estrogen receptor, *PR* Progesterone receptor, *HER-2* Human Epidermal Growth Factor Receptor-2 *AC* Adriamycin and Cyclophosphamide, *FAC* Fluorouracil, Adriamycin, and Cytoxan, *FEC* Fluorouracil, Epidoxorubicin and Cyclophosphamide *TAC* Taxotere, Adriamycin, and Cyclophosphamide *CMF* Cyclophosphamide, Methotrexate, and Fluorouracil, *TAM* Tamoxifen, *AI* Aromatase Inhibitör, *RT* Radiotherapy

**Fig. 1:**
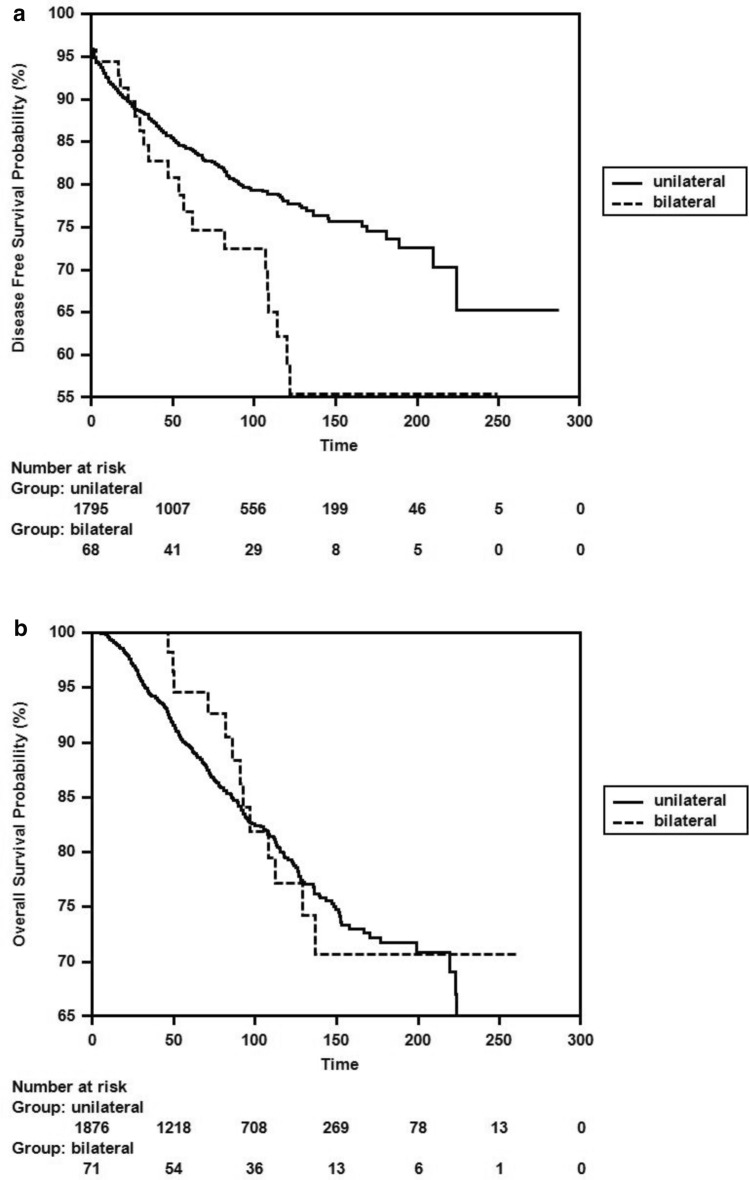
**a** Comparison of DFS duration of unilateral and bilateral breast cancer with Kaplain-Meier before propensity score analysis **b** Comparison of OS duration of unilateral and bilateral breast cancer with Kaplain-Meier before propensity score analysis

When disease-free survival (DFS) and overall survival (OS) times were calculated for breast cancer patients in our series based on whether they were in the unilateral or bilateral group, the DFS times were 220.25 ± 4.77 months in the unilateral group and 168.52 ± 13.92 months in the bilateral group (p = 0.022). The OS times were 228.37 ± 3.89 months in the unilateral group and 213.41 ± 11.80 months in the bilateral group (p = 0.859) (Fig. 1a, 1b).

When examining patient characteristics in Table 1, we recalculated the DFS and OS durations using propensity scoring to balance the statistically significant (p < 0.05) criteria identified between the two groups. The factors that showed statistical differences or were close to significance among the unilateral and bilateral breast cancer patients in our series were identified as axillary nodal status, subgroup, estrogen receptor (ER) status, Her-2 expression status, axillary surgery, and chemotherapy type. In the propensity score statistical analysis of our series, balance was achieved based on these six different characteristics. A statistical analysis was performed using data from 71 patients with bilateral breast cancer compared to 71 patients with unilateral breast cancer. After the propensity score analysis, the patient characteristics are again shown in Table 1.

**Fig. 2:**
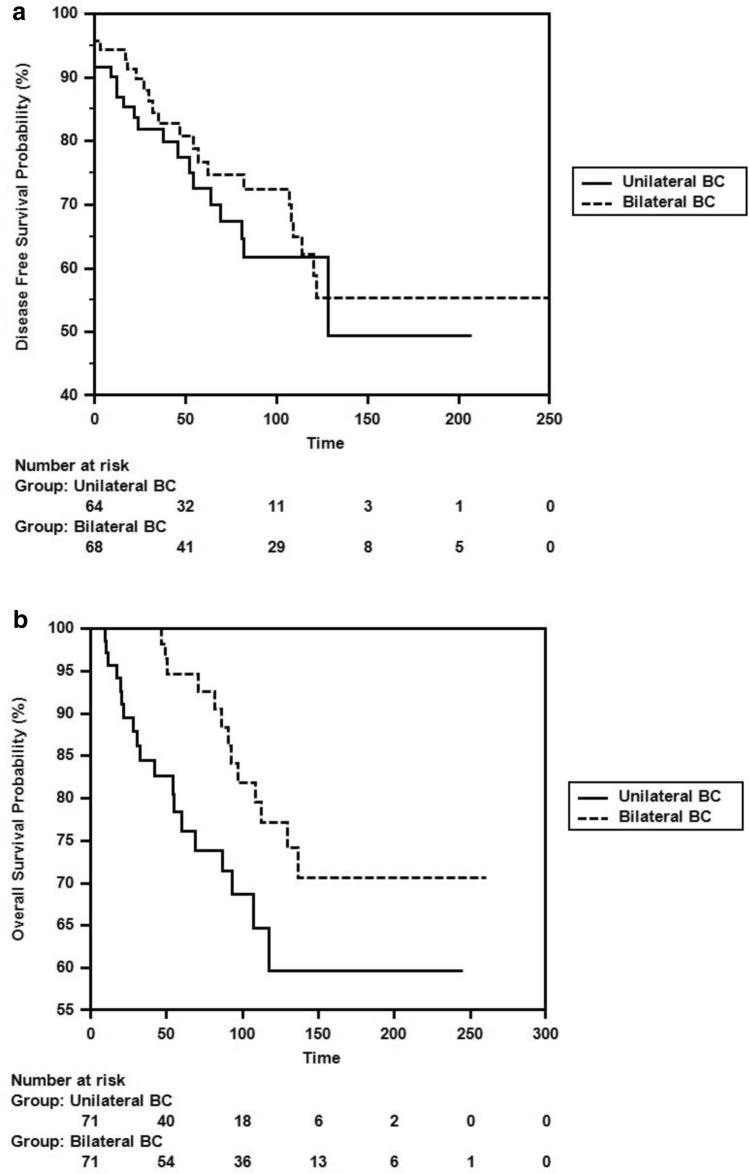
**a** Comparison of DFS duration of unilateral and bilateral breast cancer with Kaplain-Meier after propensity score analysis **b** Comparison of OS duration of unilateral and bilateral breast cancer with Kaplain-Meier after propensity score analysis

After the propensity score analysis, the DFS and OS durations calculated using the Kaplan–Meier method for patients with unilateral and bilateral breast cancer were as follows: The DFS duration was 132.24 ± 13.86 months (105.07–159.42 months) in the unilateral group and 166.86 ± 13.70 months (140.0–193.72 months) in the bilateral group (HR: 0.769, 95% CI 0.418–1.413, p = 0.397). The OS duration was 170.48 ± 14.22 months (142.61–198.36 months) in the unilateral group and 211.57 ± 11.62 months (188.78–234.35 months) in the bilateral group (HR: 0.472, 95% CI 0.232–0.963, p = 0.039) (Fig. 2a, 2b).

**Fig. 3:**
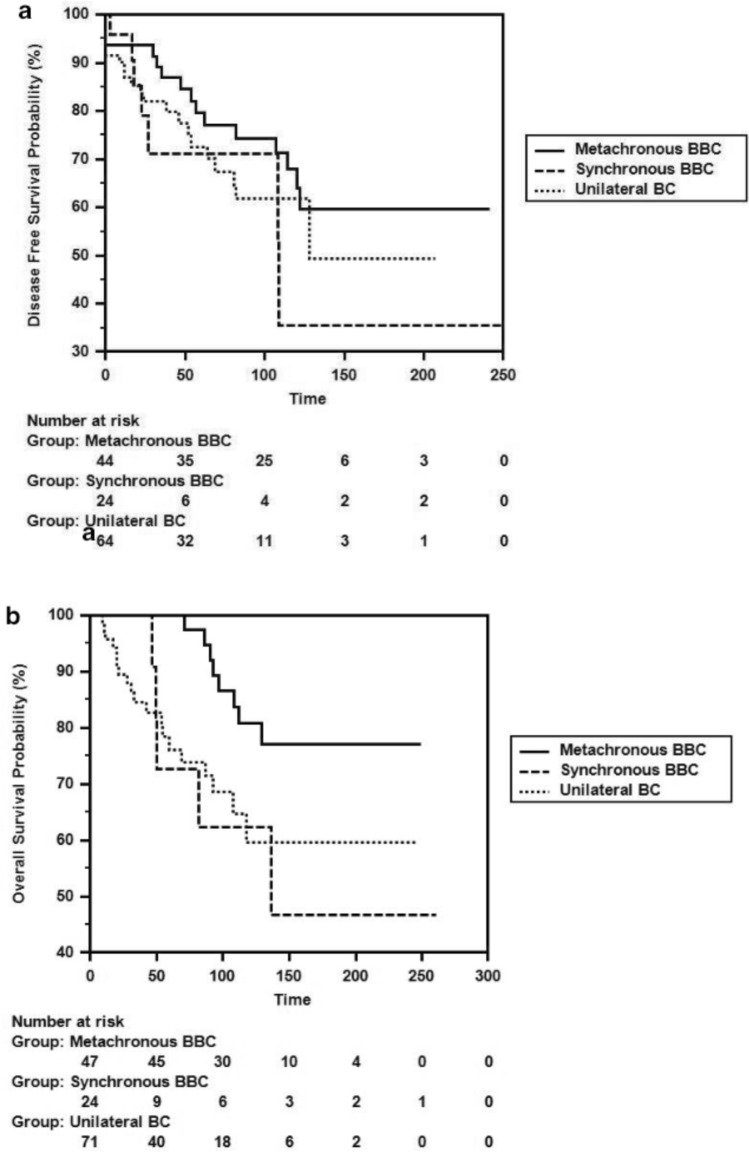
**a** Unilateral breast cancer, disease-free survival curve for synchronous and metachronous bilateral breast cancer** b** Unilateral breast cancer, synchronous bilateral breast cancer, metachronous bilateral breast cancer overall survival curve

After the propensity score balancing for patients with bilateral breast cancer, when examining the patient characteristics, we found that the following factors were statistically significantly different compared to unilateral breast cancer: a higher T stage of the tumor (p < 0.001), a greater proportion of skin involvement (p = 0.044), a lower rate of PR positivity (p = 0.011), a lower frequency of axillary surgery being axillary curettage (p < 0.001), a higher proportion of chemotherapy being adjuvant chemotherapy (p = 0.005), a greater use of FAC/FEC/TAC chemotherapy regimens (p = 0.018), a lower rate of tamoxifen usage (p = 0.039), and a higher frequency of radiotherapy application (p = 0.034) (Table 1). We separated patients with bilateral breast cancer into metachronous and synchronous groups to calculate their survival durations. The DFS durations were as follows: synchronous 132.77 ± 30.76 months (72.4–193.0 months), metachronous 170.74 ± 14.59 months (142.14–199.33 months) (p = 0.143). The overall survival durations were: synchronous 164.86 ± 30.65 months (104.78–224.94 months), metachronous 215.04 ± 10.66 months (194.13–235.94 months) (p = 0.027) (Fig. 3a, 3b).

We also examined the clinical, histopathological, and treatment characteristics of patients with metachronous and synchronous bilateral breast cancer (Table 2). Among patients with bilateral breast cancer, the number of patients with metachronous bilateral breast cancer was n = 47 (66.2%), while the number of patients with synchronous bilateral breast cancer was n = 24 (33.8%). In the bilateral breast cancer group, the most common pathology was invasive ductal carcinoma (IDC) at a rate of 61.4% (n = 43). When comparing the first tumors of metachronous and synchronous cases, there was a statistically significant difference in the N stage, with synchronous breast cancer having a more advanced N stage (p = 0.039) and a higher rate of positive surgical margins (p = 0.007). When comparing the second tumors of metachronous and synchronous cases, a statistically significant difference was found only in the higher rate of PR positivity in synchronous breast cancer (p = 0.009). Due to synchronous bilateral breast cancer having a lower survival duration within the bilateral breast cancer group, in the final step, we divided our series into three groups for comparison of survival durations: unilateral breast cancer, metachronous bilateral breast cancer, and synchronous bilateral breast cancer (Table 3). In our series, the DFS duration was 132.24 ± 13.86 months for unilateral breast cancer, 170.74 ± 14.59 months for metachronous bilateral breast cancer (p = 0.257), and 132.77 ± 30.76 months for synchronous bilateral breast cancer (p = 0.770). The OS duration was 170.48 ± 14.22 months for unilateral breast cancer, while a statistically significant difference was found in metachronous bilateral breast cancer at 215.04 ± 10.66 months (p = 0.015). When comparing unilateral breast cancer with synchronous bilateral breast cancer, the difference disappeared with a duration of 164.86 ± 30.65 months (p = 0.872) (Fig. 4a, 4b). The synchronous bilateral breast cancer (SBBC), which had the lowest OS duration, showed a statistically significant difference compared to metachronous bilateral breast cancer (MBCC), similar to that observed in unilateral breast cancer (p = 0.027) (Table 3).

**Table 2:** Comparison of clinical, histopathological and treatment features of first and second tumors of bilateral, metachronous and synchronous breast cancer patients

	Metachronous First Tumour n (%)	Synchronous First tumour n (%)	p value	Metachronous Second Tumour n (%)	Synchronous Second Tumour n (%)	p value
Histopathology IDC Non-IDC	36 (76,60) 11 (23,40)	17 (70,80) 7 (29,20)	0,598	28 (59,60) 19 (40,40)	15 (62,50) 9 (37,50)	0,245
Quandrant Site Medial Lateral Areola Multifocal	8 (17,00) 30 (63,80) 6 (12,80) 3 (6,40)	2 (8,30) 15 (62,50) 3 (12,50) 4 (16,70)	0,464	11 (23,40) 27 (57,40) 5 (10,60) 4 (8,50)	7 (29,20) 12 (50,00) 2 (8,30) 3 (12,50)	0,871
T Stage Tis T1 T2 T3 T4	0 (0,00) 14 (29,80) 24 (51,10) 5 (10,60) 4 (8,50)	0 (0,00) 9 (37,50) 13 (54,20) 1 (4,20) 1 (4,20)	0,674	6 (12,80) 23 (48,90) 13 (27,70) 2 (4,30) 3 (6,40)	3 (12,50) 15 (62,50) 5 (20,80) 0 (0,00) 1 (4,20)	0,736
N Stage N0 N1 N2 N3	24 (51,10) 6 (12,80) 5 (10,60) 12 (25,50)	13 (54,20) 5 (20,80) 6 (25,00) 0 (0,00)	***0,030***	31 (66,00) 8 (17,00) 5 (10,60) 3 (6,40)	19 (79,20) 5 (20,80) 0 (0,00) 0 (0,00)	0,203
Grade I II III	4 (8,50) 21 (44,70) 22 (46,80)	3 (12,50) 10 (41,70) 11 (45,80)	0,864	10 (21,30) 22 (46,80) 15 (31,90)	3 (12,50) 12 (50,00) 9 (37,50)	0,654
LVI	23 (48,90)	13 (54,20)	0,677	11 (23,40)	10 (41,70)	0,230
PNI	8 (17,00)	4 (16,70)	0,970	5 (0,60)	2 (8,30)	0,600
Positive Skin Involvement	3 (6,40)	2 (8,30)	0,761	4 (8,50)	1 (4,20)	0,499
Positive Surgical Margin	5 (10,60)	9 (37,50)	***0,007***	7 (14,90)	4 (16,70)	0,649
ER ( +) ER (-)	30 (63,80) 17 (36,20)	18 (75,00) 6 (25,00)	0,341	32 (68,10) 15 (31,90)	18 (75,00) 6 (25,00)	0,546
PR ( +) PR (-)	31 (66,00) 16 (34,00)	17 (70,80) 7 (29,20)	0,678	16 (34,00) 31 (66,00)	16 (66,70) 8 (33,30)	***0,009***
HER-2 ( +) HER-2 (-)	6 (12,80) 41 (87,20)	4 (16,70) 20 (83,30)	0,655	9 (19,10) 38 (80,90)	5 (20,80) 19 (79,20)	0,866
Kİ67 < %15 Kİ67 ≥ %15	11 (23,40) 36 (76,60)	2 (8,30) 22 (91,70)	0,120	23 (48,90) 24 (51,10)	9 (37,50) 15 (62,50)	0,360
Molecular Subtype Luminal A Luminal B HER-2 Enriched Triple Negative	7 (14,90) 25 (53,20) 3 (6,40) 12 (25,50)	5 (20,80) 1 (4,20) 18 (75,00) 0 (0,00)	0,208	13 (27,70) 20 (42,60) 2 (4,30) 12 (25,50)	6 (25,00) 13 (54,20) 1 (4,20) 4 (16,70)	0,787
Lymph Node Extracapsular Invasion	7 (14,90)	4 (16,70)	0,845	7 (14,90)	4 (16,70)	0,845
Surgical Type BCS MRM None Surgical	20 (42,50) 27 (57,40) 1 (2,10)	9 (37,50) 15 (62,50) 0 (0,00)	0,736	23 (48,90) 19 (40,40) 5 (10,60)	9 (37,50) 14 (58,30) 1 (4,20)	0,310
Axillary Surgery SLNB ALND None Surgery	4 (8,50) 42 (89,40) 1 (2,10)	5 (20,80) 19 (79,20) 0 (0.00)	0,272	20 (42,60) 20 (42,60) 7 (14,90)	10 (41,70) 20 (41,70) 4 (16,70)	0,981
Received Radiotherapy	39 (83,00)	20 (83,30)	0,970	37 (78,70)	16 (66,70)	0,371
Radiotherapy Type Only Breast RT Locoregional RT	13 (27,70) 26 (55,30)	7 (29,20) 13 (54,20)	0,991	16 (34,00) 21 (44,70)	9 (37,50) 7 (29,20)	0,380

*IDC* Invasive Ductal Carcinoma, *T* Tumour, *N* Nodal, *BCS* Breast Conserving surgery, *MRM* Modified Radical Mastectomy, *SLNB* Sentinel Lymph Node Dissection, *ALND* Axillary Lymph Node Dissection, *LVI* Lymphovasculer Invasion, *PNI* Perineural Invasion,, *ER* Estrogen Receptor *PR* Progesterone Receptor, *HER-2* Human Epidermal Growth Factor Receptor-2

**Table 3:** Comparison of DFS and OS times of patients with unilateral breast cancer and bilateral metachronous, bilateral synchronous breast cancer

DFS			OS		
Unilateral BC	Metachronous BBC	Synchronous BBC	Unilateral BC	Metachronous BBC	Synchronous BBC
132,24 ± 13,86 (105,07–159,42)	170,74 ± 14,59 (142,14–199,33)	132,77 ± 30,76 (72,47–193,08)	170,48 ± 14,22 (142,61–198,36)	215,04 ± 10,66 (194,13–235,94)	164,86 ± 30,65 (104,78–224,94)
*P* = 0,257	*P* = 0,143	*P* = 0,770	*P* = 0,015	*P* = 0,027	*P* = 0,872
Meachronous BBC	Synchronous BBC	Unilateral BC	Metachronous BBC	Synchronous BBC	Unilateral BC

When conducting univariate and multivariate Cox regression analyses to identify risk factors affecting DFS and OS, we identified 12 possible risk factors with p < 0.1 associated with DFS: being T4, N2 and N3 status, high-grade III (HG III), positive lymphovascular invasion (LVI), presence of skin infiltration, presence of extensive intraductal component (EIC), being ER and PR positive, type of surgery performed on the breast, timing of chemotherapy, and type of radiotherapy. In the multivariate backward stepwise Cox regression analysis considering these risk factors, the following were found to be significant for DFS: T4 stage (4.3 times), N3 stage (6.3 times), being PR positive (3.9 times).

In the univariate Cox regression analysis for OS, 10 potential risk factors (metachronous bilateral breast cancer, age, menopausal status, T4 stage, N2 and N3 stage, LVI positivity, positive skin infiltration, presence of EIC, PR positivity, type of surgery) were identified with p < 0.1. In the multivariate backward stepwise Cox regression analysis with these risk factors: N3 stage (8.1 times), presence of skin involvement (5.7 times), and ER status (2.3 times) were found to be statistically significant. When we focused on the primary objective of our study, which was the effect of bilaterality on survival, BL status did not affect disease-free survival. The presence of metachronous bilateral breast cancer reduced the risk of overall survival by 0.413 times (95% CI: 0.155 – 1.100) compared to unilateral breast cancer (p = 0.077). On the other hand, the presence of synchronous bilateral breast cancer increased the risk of OS by 2.458 times (95% CI: 0.745 – 8.110) (p = 0.140) (Table 4, Table 5).

**Table 4:** Analysis of risk factors affecting DFS and OS duration according to clinical, histopathological and treatment characteristics of patients with unilateral and bilateral breast cancer using univariate cox regression analysis

	DFS		OS	
Patient Characteristics	p	HR 95% CI (Lower–Upper)	p	HR95% CI (Lower–Upper)
Unilateral BC Bilateral BC	0,397	0,769 (0,41–1,41)	**0,039**	**0,47 (0,23–0,96)**
Unilateral BC Metachronous BBC Synchronous BBC	1 0,22 0,712	Reference 0,654 (0,33–1,28) 1,176 (0,49–2,77)	1 **0,016** 0,896	Reference **0,358 (0,15–0,82)** 0,936 (0,34–2,51)
Age	0,332	1,014 (0,98–1,04)	**0,001**	**1,05 (1,02–1,09)**
Menopausal Status	0,331	1,382 (0,72–2,65)	0,062	2,22 (0,96–5,15)
Family History	0,189	0,622 (0,30–1,26)	0,913	1,04 (0,49–2,20)
Histopathology	0,261	0,644 (0,29–1,38)	0,671	1,18 (0,54–2,55)
Tumor Quadrant Lateral Medial Areola Multifocal	1 0,583 0,428 0,297	Reference 1,267 (0,54–2,94) 1,406 (0,60–3,26) 1,672 (0,63–4,39)	1 0,574 0,302 0,106	Reference 0,704 (0,20–2,39) 1,628 (0,64–4,10) 2,270 (0,84–6,13)
BBC Tumor Quadrant Lateral Medial Areola Multifocal	1 0,113 0,557 0,625	Reference 0,361 (0,10–1,27) 0,542 (0,07–4,17) 1,371 (0,38–4,86)	1 0,667 0,987 0,439	Reference 0,747 (0,19–2,82) 0,00 (0,00–0,00) 1,845 (0,39–8,70)
T Stage T1 T2 T3 T4	1 0,235 0,767 **<0 ,001**	Reference 1,796 (0,68–4,72) 1,242 (0,29–5,19) **10,240 (3,46–30,28)**	1 0,339 0,382 **< ,001**	Reference 1,83 (0,52–6,40) 2,04 (0,41–10,12) **11,24 (3,12–40,47)**
N Stage N0 N1 N2 N3	1 0,602 **0,01** **< 0,001**	Reference 1,349 (0,43–4,15) **3,192 (1,31–7,75)** **5,481 (2,64–11,35)**	1 0,789 **0,001** **< 0,001**	Reference 1,23 (0,25–5,97) **5,31 (1,91–14,74)** 7,78 (3,15–19,21)
Histological Grade I II III	1 0,247 0,076	Reference 3,308 (0,43–25,09) 6,135 (0,83–45,36)	1 0,318 0,281	Reference 2,80 (0,37–21,27) 3,03 (0,40–22,94)
Positive LVI	**0,018**	**0,477 (0,25–0,88)**	**0,013**	**0,39 (0,18–0,81)**
Positive PNI	0,571	0,808 (0,38–1,68)	0,999	1,01 (0,41–2,43)
Positive Surgical Margine	0,302	1,479 (0,70–3,11)	0,58	1,28 (0,52–3,14)
Positive Skin Infiltration	**< 0,001**	**0,218 (0,10–0,45)**	**<0 ,001**	**0,12 (0,05–0,25)**
Positive Extracapsular Invasion	**< 0,001**	**0,289 (0,14–0,56)**	**< 0,001**	**0,25 (0,12–0,51)**
Negative ER	**0,054**	**1,802 (0,99–3,28)**	0,131	1,70 (0,85–3,41)
Positive PR	**0,004**	**2,50 (1,33–4,70)**	**0,077**	**1,89 (0,93–3,83)**
< Ki67, ≥ Ki67	0,393	1,56 (0,55–4,40)	0,112	5,04 (0,68–36,95)
Positive HER2	0,463	0,722 (0,30–1,72)	0,725	0,82 (0,28–2,37)
Molecular Subtype HER2 Enriched Triple Negative Luminal A Luminal B	1 0,327 0,914 0,59	Reference 1,847 (0,54–6,29) 1,070 (0,31–3,60) 1,638 (0,27–9,84)	1 0,601 0,202 0,47	Reference 0,667 (0,14–3,03) 0,209 (0,01–2,31) 0,583 (0,13–2,52)
Surgery Type	**0,001**	**3,174 (1,55–6,46)**	**0,001**	**5,03 (1,93–13,07)**
Time to Administer CT (Adjuvant CT vs Neoadjuvant CT)	**0,027**	**0,443 (0,21–0,91)**	0,285	0,613 (0,25–1,50)
Received Transtuzumab	0,708	0,820 (0,29–2,31)	0,877	1,121 (0,26–4,74)
TAM Use Duration (< 5 year vs ≥ 5 year)	0,619	0,590 (0,07–4,72)	0,423	0,037 (0,00–115,99)
AI Use Duration (< 5 year vs ≥ 5 year)	0,95	0,969 (0,36–2,57)	0,184	0,422 (0,11–1,50)
RT Type None RT Only Breast RT Locoregional RT	**1** ***0,016*** 0,598	**Reference** **0,271 (0,09–0,78)** 0,809 (0,36–1,77)	**1** 0,774 0,137	**Reference** 1,265 (0,25–6,29) 2,997 (0,70–12,72)

*BC* Breast cancer, *BBC* Bilateral breast cancer, *IDC* Invasive Ductal Carcinoma, *T* Tumour, *N* Nodal, *BCS* Breast Conserving surgery, *MRM* Modified radical mastectomy, *SLNB* Sentinel lymph node biopsy, *ALND* Axillary lymph node dissection, *LVİ* Lymphovasculer invasion, *PNİ* Perineural invasion, *ER* Estrogen receptor, *PR* Progesterone receptor, *Her-2* Human epidermal growth factor receptor-2, *CT* chemotherapy, *TAM* tamoxifen, *AI* aromatase inhibitör *RT* radiotherapy

**Table 5:** Analysis of risk factors affecting DFS and OS duration according to clinical, histopathological and treatment characteristics of patients with unilateral and bilateral breast cancer using multivariate backward stepwise cox regression analysis

DFS	*p*	HR 95% CI (Lower–Upper)	OS	*p*	HR 95% CI (Lower–Upper)
T Stage T1 T2 T3 T4			Bilaterality Status		
	1	Reference	Unilateral BC	1	Reference
	*0,844*	1,118 (0,367–3,409)	Metachronous BBC	***0.077***	**0.413 (0.155–1.100)**
	*0,924*	1,077 (0,235–4,941)	Synchronous BBC	0.140	**2.458 (0.745–8.110)**
	***0,028***	**4,296 (1,171–15,762)**	**N Stage**		
N Stage			N0	1	Reference
N0 N1 N2 N3	1	Reference	N1	0.875	1.136 (0.231–5.591)
	0,223	2,368 (0,592–9,480)	N2	0.814	1.179 (0.299–4.652)
	*0,114*	2,279 (0,820–6,334)	N3	***0.001***	**8.136 (2.486–26.624)**
	**< *0,001***	**6,314 (2,403–16,595)**	Positive Skin Infiltration	***0.001***	**0.175 (0.064–0.481)**
**ER**	*0,110*	0,454 (0,172–1,196)	Positive ER	***0.051***	**2.277 (0.995–5.211)**
**PR**	***0,001***	**3,859 (1,682–8,857)**	Surgery Type	***0.075***	**2.817 (0.901–8.807)**

## Discussion

The rate of bilateral breast cancer in our series was 3.6%, with MBBC constituting 2.4% and SBBC 1.2%. Our incidence of bilateral breast cancer, particularly SBBC, was comparable to those reported in the Western series [7–9]. Regarding synchronous bilateral breast cancer, DFS duration was similar to MBBC and unilateral breast cancer, while it was the breast cancer subtype with the shortest overall survival time. In our study, patients with metachronous bilateral breast cancer (MBBC) had a statistically significantly longer survival time compared to those with unilateral breast cancer. The overall survival (OS) durations in our series ranked as follows: MBBC had the longest survival, followed by unilateral breast cancer, and then synchronous bilateral breast cancer (SBBC). Additionally, there was a statistically significant difference in survival time between SBBC and MBBC (p = 0.027). Consequently, our findings did not support previous reports suggesting that bilateral breast cancer has a similar or better survival time compared to unilateral breast cancer [2, 9].

In the histopathological, clinical, and treatment characteristics of the patients in our series, after propensity score analysis, it was found that unilateral BC had a more advanced tumor stage (p < 0.001), a higher rate of skin involvement (p = 0.044), leading to a higher rate of neoadjuvant chemotherapy (p = 0.005), and a higher rate of receiving radiotherapy (p = 0.034). In the bilateral BC group, the statistically significant differences included a higher rate of PR positivity (p = 0.011), a higher rate of axillary curettage (p < 0.001), and a greater use of tamoxifen (p = 0.039). In the comparison of the primary tumors of patients with BBC, categorized as MBBC and SBBC, we observed a more advanced N stage in metachronous BBC (p = 0.030), a higher rate of positive surgical margins in SBBC (p = 0.007), and a higher rate of progesterone receptor (PR) positivity in the secondary tumors of SBBC (p = 0.009). Despite the more advanced N stage in MBBC, the longest survival time was observed. At the same time, SBBC had the shortest survival time, which was notable for its association with positive surgical margins and higher PR positivity. It was particularly challenging to explain the lowest survival rate observed in SBBC based on patient and treatment characteristics. A study by Kwast et al. suggested that SBBC might have an undiscovered genetic mutation, which could be significant in the cancer’s different progression. They reported that SBBC’s tendency toward tertiary cancers outside the breast could indicate that it is a distinct subtype of cancer [19].

In the literature review, studies supporting the results of our series [4, 7, 8] also reported that the overall survival (OS) time in patients with SBBC is shorter. As Holm M. et al. stated in their meta-analysis, SBBC is associated with the lowest OS, and they propose that, even though these patients may have smaller and less aggressive tumors, the presence of SBBC should be considered a poor prognostic factor [7, 8]. Additionally, Jobsen JJ et al. noted that synchronous tumors are less frequently observed. While this rate may increase with modern imaging techniques, the preference of patients with unilateral BC for bilateral mastectomy as a surgical option may reduce this likelihood [8].

In our series, while the bilateral status did not affect DFS in the multivariate Cox regression analysis when bilateral cancers were collectively compared to unilateral breast cancer for OS, it had a risk-reducing effect (by 2.1 times). When the same analysis was conducted by comparing MBBC and unilateral BC, we observed that the risk of death was reduced by 2.8 times, which was close to statistical significance. However, when SBBC was compared to unilateral BC, the risk of death increased by 2.5 times, although this increase was not statistically significant. The much better survival observed in metachronous breast cancer compared to unilateral breast cancer can be attributed to the fact that these patients are already under follow-up and, with the diagnosis of the secondary tumor, they re-enter a strict follow-up process.

In our series, risk factors affecting DFS (T4 stage, N3 stage, being PR positive) and OS (N3 stage, skin involvement, being ER positive) were identified through multivariate Cox regression analysis. However, risk factors for BBC mentioned in the literature, such as a family history of breast cancer, young age at first diagnosis, and having lobular carcinoma, were not identified as risk factors in our series. Another risk factor mentioned in the literature is the increased risk of developing BBC in patients with germline mutations in the BRCA1 or BRCA2 genes [2, 7, 20]. In our series, a germline mutation in the BRCA1 or BRCA2 genes could not be statistically analyzed due to the limited number of patients tested for it. As noted by Mruthyunjayappa S. and colleagues, since the prevalence of BRCA1 and BRCA2 mutations is very low and the tendency for prophylactic bilateral mastectomy in healthy women and prophylactic contralateral mastectomy in women with UBC is increasing, most BBC patients do not carry these mutations [2, 9].

When examining Figs. 3a and 3b in our series, 75% of SBBC patients experienced recurrence/metastasis in a shorter period, and 62.5% of the patients were lost within the first 5 years. The finding that “bilaterality has no negative effect on survival.” As mentioned in studies where SBBC and MBBC are evaluated together [2, 9, 10, 19], was not surprising. Perhaps the fact that we are dealing with a subgroup with a lower incidence compared to unilateral breast cancer may have caused an imbalance in the comparisons. However, it is possible to say that propensity score analysis, a statistical method, is a golden statistical analysis method designed precisely for the evaluation of groups with numerical imbalances, such as in this case [15, 16].

There are currently no standard treatment guidelines for BBC treatment. Therefore, developing a personalized treatment plan is done by considering the patient’s age, histological subtype of the tumor, and tumor stage. Although the incidence of BBC differs between Western and Eastern countries, we see that treatment strategies, especially surgical approach options, are similar. Chen, JJ et al. [21, 22] reported that bilateral mastectomy was the primary surgical treatment option, particularly for SBBC patients, which was consistent with the findings in our series. Similarly, in the study by Beckmann et al., the number of patients who underwent mastectomy and ALND in BBC was higher, while the number of patients receiving RT was lower [23, 24]. In our study, while the surgical findings were similar, the number of patients receiving RT was higher.

In our study, the rate of axillary lymph node dissection (ALND) was 45.1% for unilateral breast cancer and 85.9% for bilateral breast cancer. More aggressive surgical approaches were preferred in the bilateral breast cancer group in terms of both local and axillary surgery. However, when comparing the T stage and N staging of unilateral breast cancer patients to bilateral breast cancer patients after propensity score analysis, the unilateral breast cancer group was found to be at a more advanced stage (Table 1). For this reason, the neoadjuvant chemotherapy option was more frequently preferred in patients with unilateral breast cancer (23.9% in unilateral breast cancer, 11.3% in bilateral breast cancer, p = 0.005).

In the series by Jia H. et al., the luminal B type was the most observed group in the bilateral breast cancer group [3]. In our series, Luminal B was also the most frequently seen subtype. Similarly, in the study by Kollias et al., which had results like ours, high-grade tumors constituted 29% of metachronous tumors, while they made up 45% and 54% of unilateral and synchronous tumors, respectively. In SBBC, tumors with a diameter ≥ 2 cm had lymph node involvement [12]. In our series, the rate of grade III was 39.4% for MBBC, 47.9% for unilateral BC, and 41.7% for SBBC. Notably, the rate of positive surgical margins in our series was higher in patients with synchronous breast cancer. This finding is significant because a considerable number of patients chose mastectomy, and both breasts were operated on simultaneously. This approach aimed to minimize surgical morbidity for both the surgeon and the patient, as well as to prevent delays in the initiation of adjuvant therapy.

Although international evidence regarding the prognostic significance of synchronous bilateral breast cancer is inconsistent, survival rates for these patients are generally found to be similar to or worse than those for unilateral breast cancer [7, 8, 23–27]. To better understand the reasons for the poor prognosis, researchers propose that undiscovered genetic factors may contribute, acknowledging that the low incidence rate of approximately 1% may prevent large-scale studies or randomized trials. Consequently, future research should focus on exploring these potential genetic factors [19].

Despite the small number of patients, when matched with propensity score analysis, the patient group with the lowest overall survival was the synchronous bilateral breast cancer group. Due to the wide variability in clinical and histopathological features and the time elapsed between the diagnosis of the first and second tumors [28], it has not been possible to conduct a risk analysis for synchronous bilateral breast cancer. Therefore, meticulous planning and modification of surgical and adjuvant treatment for patients with synchronous bilateral breast cancer is crucial. The discovery of genetic factors that may be identified in the coming years will also be particularly important for this patient group.

The limiting factors in our study were the retrospective nature of our series and the lack of information on BRCA-1 and BRCA-2 mutations in our patients. Although there are studies suggesting that survival outcomes for breast cancer patients with BRCA-1 or BRCA-2 mutations may be worse, similar, or better compared to non-carriers, none of the patients in our study had mutation status available for propensity score analysis, and therefore, we could not include this information in our analysis. In studies analyzing patients with BRCA1 and BRCA2 mutations, Lee et al. found that BRCA1 mutations decreased overall survival (OS) and progression-free survival (PFS), while BRCA2 mutations did not. On the other hand, Zhong et al. suggested that BRCA1 mutations were associated with worse OS, but not with PFS, and that BRCA2 mutations were not linked to either worse OS or PFS. They noted that the contradictory nature of these findings was likely due to limited statistical power [29–31]. However, in recent years, particularly with younger patients, we have been able to identify individuals with BRCA1 and BRCA2 mutations and have had the opportunity to provide genetic counseling, similar to the study by Liu et al. [32]. Therefore, we believe that future studies will allow us to share this information as well.

## Conclusion

The fact that SBBC has the lowest survival rate despite more aggressive treatments should be considered a poor prognostic factor for survival on its own. Current treatment guidelines are insufficient for SBBC due to characteristics that remain unknown to us. Therefore, SBBC should be evaluated as a separate subgroup. The time elapsed from the initial diagnosis to the diagnosis of secondary breast cancer (0–12 months) needs clarification regarding which subgroup the BCC belongs to. This clarity in timing will facilitate determining the treatment and follow-up process for MBBC and SBCC. Since a common language will emerge in future studies, the similarities and differences of the series will be better understood.

## Data Availability

No datasets were generated or analysed during the current study.
